# Can a monologue-style ECA more effectively motivate eHealth users in initial distress than textual guidance?

**DOI:** 10.1016/j.heliyon.2021.e06509

**Published:** 2021-03-21

**Authors:** Mark R. Scholten, Saskia M. Kelders, Julia E.W.C. Van Gemert-Pijnen

**Affiliations:** a Department of Psychology, Health and Technology, Centre for eHealth and Wellbeing Research, University of Twente, Enschede, Netherlands; b Optentia Research Focus Area, North-West University, Vanderbijlpark, South Africa

**Keywords:** Stress, eHealth, Support, Embodied conversational agent, Affective computing, Persuasive technology

## Abstract

Stress is a prevalent issue amongst patients with chronic conditions. As eHealth interventions are gaining importance, it becomes more relevant to invoke the possibilities from the eHealth technology itself to provide motivational acts during experiences of stress as to enhance adherence to the intervention. Embodied Conversational Agents (ECA's) also known as ‘robots on screen’ can potentially provide a remedy. Within our eHealth experiment we applied a between-subjects design and experimentally studied the difference in appraisal of motivation and guidance. We deployed a functionally modest, monologue-style ECA and compared them with textual guidance. This way, we filtered out the considerable positive impact of interactive features that go along with dialogue-style ECA's. The study was carried out amongst eHealth users of which half were deliberately put in a stressful pre-condition. The rationale was two-sided; first, we hypothesized that it would induce a need for motivational support. Second, it would provide a fair representation of eHealth users in real life. Furthermore, we investigated hypothesized positive effects from a gender match between participant and ECA. The results demonstrated preferential ECA effects compared to text but only in the no stress conditions. Although our set-up controlled for user distraction by putting the facilitating ECA in a pane separate from the eHealth environment, we suspect that the enduring visual presence of the ECA during task completion had still inhibited distressed users. Discussing this phenomenon, our stance is that the hypothesis that ECA support is always superior to textual guidance is open for re-evaluation. Text may sometimes serve users equally well because it lacks *human-like* aspects that in stressful circumstances can become confrontational. We discuss the potential of ECA's to motivate, but also elaborate on the caveats. Further implications for the ECA, intervention adherence, and eHealth study fields are discussed in relation to stress.

## Teaser

Stress; eHealth; Support; Embodied conversational agent; ECA; Persuasive technology

## 1 Introduction

It is well-established finding (see e.g. Vancampfort et al., 2017) that patients with chronic health conditions face elevated levels of stress. Stress is broadly defined as “a process by which a challenging emotional or physiological event or series of events result in adaptive or maladaptive changes required to regain homeostasis and/or stability” (Sinha and Jastreboff, 2013). Probably the most prominent physical cause of stress is pain (Abdallah and Geha, 2017; Rosenzweig et al., 2010). Stress amongst patients is also induced in more indirect ways such as the patient's dwellings on his or her long-term prognosis. In Vancamfort et al.'s (2016) large epidemiological study on data from 229,293 adults living in 44 countries it is described in detail how chronic conditions lead to stress and reversely how stress worsens chronic conditions. Furthermore, the authors describe that stress can intensify the effect of chronic diseases such as asthma, arthritis, or diabetes as it increases experiences of pain and decreases adherence to medical treatment protocols. Within the eHealth domain, defined as ‘the use of information and communication technologies (ICT) for health’ (World Health Organization, 2016), stress is also referred to as a relevant factor. Leenen et al. (2016) describe eHealth patients' stressful experiences in relation to their diseases in their study. As reported by the authors, carrying out eHealth self-management tasks is perceived by patients as an encounter with their physical and mental states. In a similar vein, Huygens et al. (2016) state that eHealth patients can become anxious from the information they find, particularly when reading information about complications that could occur at a later stage of their disease. But also carrying out seemingly innocent daily practical eHealth tasks can have unexpected stressful effects. Huygens et al. (2016) refer to a patient's story measuring blood data as a routine, becoming more aware of his condition, and ultimately notifying this as a highly unpleasant experience. Another germane study (Kelders et al., 2013) reports on a group of users who dropped out from an intervention designed to reduce depressive complaints. This withdrawal occurred after a lesson that focused on the application of newly acquired skills in practice. Apparently, this lesson turned out to be too confrontational. Note that -from a treatment perspective-this lesson was as a key event for reaping the benefits from the eHealth intervention. Altogether, these studies suggest that eHealth self-management -although a sensible activity from a medical perspective- is often a daunting task from an emotional and personal perspective. In such as stressful situation, many patients lose motivation to continue using their eHealth interventions. Stated differently, intrinsic patient motivation starts to wane and external support has to be invoked.

### 1.1 Persuasive technology providing user support

A remedy to stimulate a patient's motivation is offered by persuasive technology. Persuasive technology is defined as ‘computerized software or information systems designed to reinforce, change or shape attitudes or behaviors or both without using coercion or deception’ (Oinas-Kukkonen and Harjumaa, 2009). According to Van Gemert-Pijnen et al. (2018) persuasive technology is characterized by increased interactivity and engagement of users through modern information and communication technologies. A relevant instance of persuasive technology is the Embodied Conversational Agent, abbreviated as ECA. An ECA is a more or less autonomous and intelligent software entity with an embodiment used to communicate with the user (Ruttkay et al., 2004). Encouraging experimental set-ups have been realized with ECA's concerning the promotion of healthy behavior amongst patients (Sillice et al., 2018), training aspiring doctors for emotionally charged encounters with patients (Kron et al., 2017) and reaching out to a population that has an elevated Post Traumatic Stress Syndrome (PTSS) profile but is avoiding mental healthcare (DeVault et al., 2014).

### 1.2 The present state of the ECA study field

Although these ECA studies hold promise, Weiss et al. (2015) has convincingly outlined both the complexity and subtlety of the ECA study field. As Weiss et al. (2015) point out; depending on the application domain, different performance and quality aspects are important. That is, in a health literacy context, the ECA is required to engage the user. In contrast, in a care-taking situation, conveying empathy and provoking emotions are apt. With regards to their evidence, several meta-analyses have evaluated ECA effects, mostly within the eLearning domain. Within the meta-analysis of Schroeder et al. (2013) on 43 studies including 3,088 participants, a small but significant effect is reported on learning. The participants learned more from a system with an ECA, than a system without one. A second meta-analysis (Veletsianos and Russell, 2014) reports on studies in which both motivation and learning outcomes are promoted by ECA's. However, the authors also refer to studies on ECA's that failed to demonstrate added value compared to text-only conditions. Veletsianos and Russell (2014) summarized these mixed results as a conundrum and a challenge for new studies to take up.

### 1.3 This study as a successor of earlier positive ECA results

Within our earlier study (Scholten et al., 2019) we deployed a monologue-style male ECA as an adjunct in an eHealth psycho-education intervention and compared its impact to a textual guidance control condition. The eHealth psycho-education intervention was devised to improve the mental well-being of its users, by teaching positive psychology concepts. The ECA and textual conditions displayed almost precisely (verbatim) the same, non-interactive guiding and motivational information as to ensure a fair comparison. The only additional information that the ECA provided, was stating his name. The intervention taught positive psychology theory and provided exercises and most participants reported it as a pleasant experience. We found a positive effect of the ECA's task-related, practical support. In contrast, we didn't find a user preference for the ECA because of its emotion-related, motivational capabilities. Following up on these results within this present study, we raise several topics.

As a first follow-up question on the Scholten et al. (2019) study: could the gender of the (male) ECA have played a role in the evaluation of the (mostly female) participants? Stated differently, could a match in gender between ECA and participant have contributed to a more positive user assessment? As reviewed in Baylor (2009), learners tend to be more influenced by an ECA of the same gender and ethnicity than agents who differ in those respects. Note that this phenomenon is similarly found in a human to human context; people are more readily persuaded by members of their in-group. Richards et al. (2020) reported on users preferring someone from their own age or older but either having no preference for gender and ethnicity or preferring their own gender and ethnicity. Ranjbartabar and Richards (2019) sketched an even more detailed picture with users preferring similarity of the ECA in terms of age, gender and culture when the ECA takes on a supportive role. However, in case the ECA's purpose is to challenge and change biases, dissimilarity with the user is acceptable and sometimes even preferable. Whatever is most beneficial in achieving the goal should be implemented, as is demonstrated by Ranjbartabar and Richards (2019). They describe an ECA representing an elderly person. This senior ECA explains effectively what it is like to be someone of age. It is readily accepted, despite its obvious dissimilarity with the age of the young study participants. Nevertheless, following the mainstream of experimental evidence on similarity, we hypothesize that a young ECA in our new experimental set-up will result in enlarged support effects amongst young study participants with the same gender.

As a second follow-up topic, the relationship between user and ECA needs to be further investigated. As we know from the literature (Bickmore and Picard, 2005), support that is provided by an ECA that has priorly established a relationship with the user has a much higher chance of being effective than support from an unfamiliar ECA. The quality of this user-ECA relationship is usually measured by the construct of rapport (Scholten et al., 2017). Rapport has to do with a positive working relationship and being ‘in tune’ or ‘click’ with each other. The role of rapport in fostering effective social human interactions is well established. As reported by Gratch et al. (2013), rapport is underlying processes as diverse as social engagement (Tatar, 1997), success in teacher–student interactions (Bernieri, 1988), productive negotiations (Drolet and Morris, 2000), psychotherapeutic effectiveness (Tsui and Schultz, 1985). ECA's have been created that make use of small talk and humor as relationship building techniques (Bickmore, 2010). Some of these ECA's present themselves as mere speakers, thereby smartly avoiding the risk of falling short on their communication capabilities. In those cases, ECA's introduce themselves to the participant and explain their roles as support providers. This personal introduction -which is common practice within a human to human context-effectively creates a base of rapport between user and ECA (Bickmore, 2010). Note that such a personal introduction cannot be credibly provided through mere text, as there is not a visible sender as a source and point of reference. So, the visibility and personality of the ECA gives it distinctive qualities compared to textual guidance.

As a third follow-up topic, we hypothesize that study participants in distress are more in need of support than the participants in the original Scholten et al. (2019) study. In other words, stressed eHealth intervention users potentially value the supportive ECA better. Moreover, an experimental set-up including stress as a factor, makes it a more life-like eHealth intervention. Note however that empirical studies on ECA support for participants under stress are scarce. Prendinger et al. (2005) indicated that their affective ECA reduced the stress of participants as measured by galvanic skin response, and also led participants to experience a quiz as less difficult. Another study (Sanghoon and Roberto, 2005) showed that the ECA's presence led to extra user stress. Thus, at first sight their ECA was counter-productive. However, as the authors concluded, the ECA's presence could still be considered as beneficial as it ultimately helped the user venting their stress experience. Ranjbartabar et al. (2019) deployed a neutral and empathic virtual therapist (ECA) in combination with a relaxation technique called Emotional Freedom Technique (EFT). Both types of ECA's were offered to the user, but in different order. Participants who were facing emotional issues built more rapport with the ECA that immediately showed empathy, which held when the ECA later became neutral. In contrast, participants that lacked these emotional issues displayed larger rapport with the ECA that initially acted neutrally. In short their study showed that the ECA's behavior should accommodate to the user's emotional state in order to be accepted by the user. Highly relevant for our study, their study also demonstrated that users who are in distress value emotional support better than users who are in a neutral state. An important sidenote has to be made here. It is often assumed that guidance by an ECA is superior to textual guidance. However, this does not always prove to be the case. Ranjbartabar and Richards (2018) deployed two conditions of an intelligent virtual advisor (ECA) with the purpose of reducing study stress and compared them to a textual support condition (a pdf document). They deployed an adapted rapport scale in which the rapport with the textual document was measured (e.g. “I felt had a connection with the document”). As their results showed, groups that were supported by the ECA reported significantly lower levels of study stress than the textual condition. However, their results also unexpectedly showed that the textual condition led to *higher* rapport scores than the ECA conditions, although the effect did not reach significance. A last relevant study to mention in relation to stress and ECA's is that of Blankendaal et al. (2015). They deployed an ECA to create user frustration. Their results showed that their ECA was successful, but to a smaller extent than that of a condition with a frustration generating human.

For the purpose of this study we investigate whether this distinctive rapport creation ability will result in a user preference effect towards the supportive ECA. In addition, we aim to assess whether the ECA's rapport building activities will transfer to an overall positive eLearning experience. Such findings have been found before by Karacora et al. (2012) in their study on outcomes of math puzzles. Participants who were interacting with the agent that built rapport showed a significantly higher improvement in learning performance than participants who interacted with an agent that lacked rapport-building skills. Moreover, we intend to investigate whether this effect will enlarge for users under stressful circumstances. Our underlying assumption is that stress will lead to an enlarged user need and appreciation for external support as provide by the ECA. As a precondition for effective support the literature tells us that ECA's should be capable of credibly presenting themselves as solutions for stress and avoid to be regarded as an additional source of user stress. As mentioned before, studies have shown that this can be achieved through the creation of a basic level of rapport with the user. However, it is an open question whether rapport will hold in stressful circumstances and whether the ECA will remain to be an effective support provider. We will therefore specifically address these matters within the present study. Furthermore, note that many rapport building ECA's (see e.g. Gratch et al., 2013) deploy dialogue and their effects are compared with those of humans. Within our present study, we take another perspective; we will compare monologue-style, low-tech ECA's with textual support. The rationale behind this approach is that dialogue features are salient and will often conceal the smaller effects of the ECA's mere visual presence. By deploying a monologue-style ECA, the dialogue effect is left out of the equation, and the smaller effects will be more likely become distinguishable. Furthermore, we will be able to test the hypothesis that ECA guidance is superior to textual guidance and support in case eHealth users are in distress.

### 1.4 This study

In this study we will include stress-induction on users, vary the ECA's gender, and stimulate the creation of rapport. We will verify the effects on the appreciation of the ECA. This brings us to the following research questions:

1) To what extent can we find preferential effects for the monologue-style ECA compared to text, as to replicate the effect of the Scholten et al. (2019) experiment?
2) To what extent does the experience of user distress positively affect the evaluation of the monologue-style ECA?
3) To what extent do eHealth users provide higher ECA evaluations when interacting with a monologue-style ECA of the same gender as compared to a monologue-style ECA of different gender?
4) To what extent do positive user evaluations of the monologue-style ECA lead to higher involvement of the user with the eHealth intervention?

Altogether, with the research questions we expect to find substantiation for monologue-style ECA's as effective eHealth support providers. Stated differently, we aspire to find that the results corroborate the promise that monologue-style ECA adjuncts provide a potential remedy for experiences of stress amongst users of self-guided eHealth interventions.

## 2 Materials and methods

### 2.1 Recruitment of participants

We recruited bachelor and master psychology and communication students at the University of Twente. As an inclusion criterion we set proficiency in English. As an exclusion criterion we set participation in a previous study with the ECA. The study protocol was reviewed and approved by the Twente Institutional Review Board, the Behavioral and Management Sciences Ethics Committee under number 190801. In total 106 participants were included. All participants gave informed consent in accordance with the Declaration of Helsinki. Participants were on average 20.4 years of age and represented 15 nationalities of which German (69%) and Dutch (18%) were most prominent. 80 participants were female (75.5%), 26 participants male (24.5%).

### 2.2 Design

To investigate the differential outcome effects of monologue-style ECA's as a result of inducing stress and of a matching gender effect using a between-subjects design we set up the following pre-conditions and factors:

• Stressful versus non-stressful pre-condition (2 pre-conditions)
• Male ECA, female ECA, textual guidance (3 factors)

This resulted in 2∗3 = 6 combinations to which participants were randomized.

**Figure 1:**
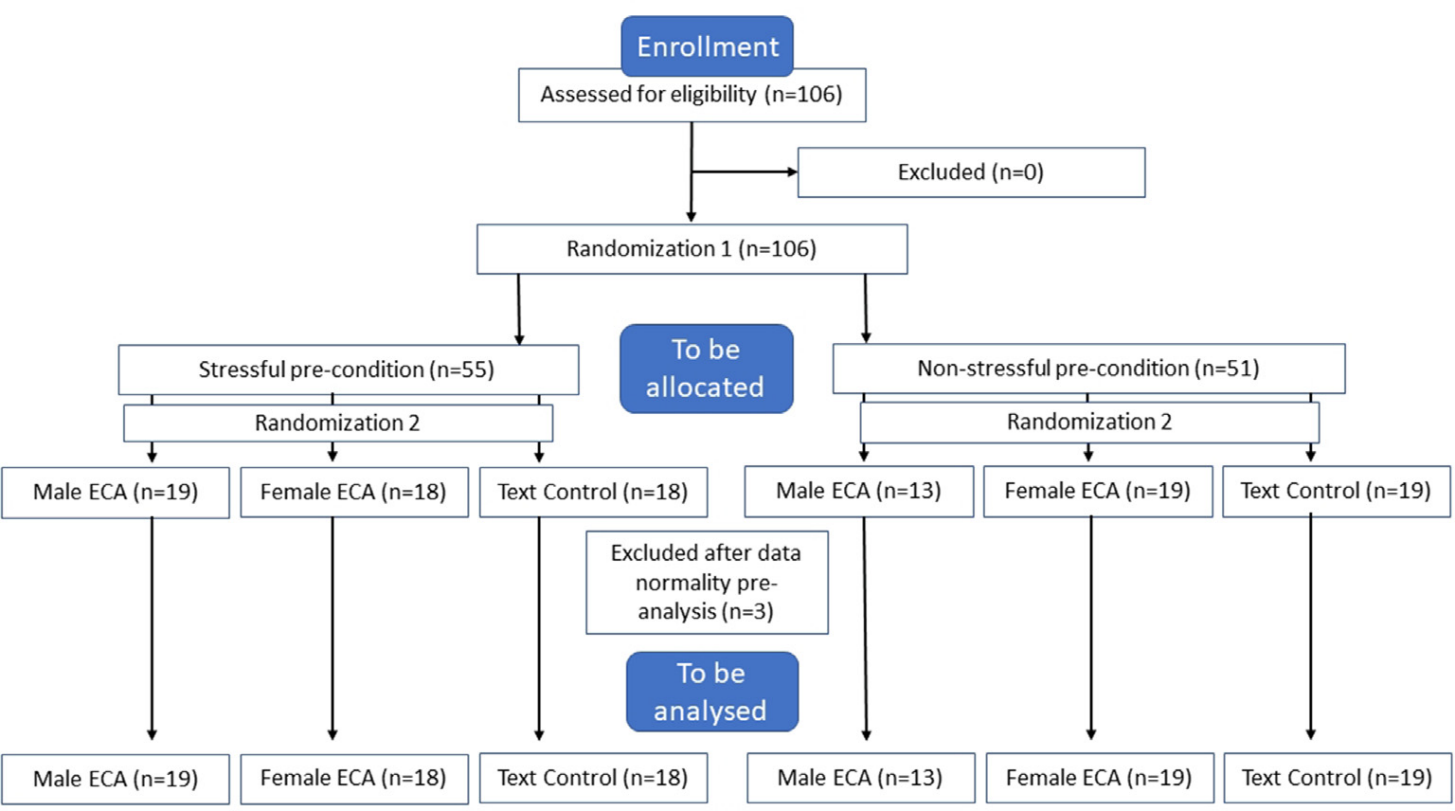
CONSORT flow chart of the study participants.

The study design was a between-subjects experiment with two factors: the stress factor with 2 levels and the support factor with 3 levels. As portrayed in Figure 1 below, randomization was done in two steps: during the first randomization, participants were either assigned to a stress or no stress pre-condition. During the second randomization, the participants were assigned to an e-learning intervention with as guidance either a female ECA, a male ECA, or text (control condition).

### 2.3 Intervention

#### 2.3.1 Pre-conditions

**Figure 2:**
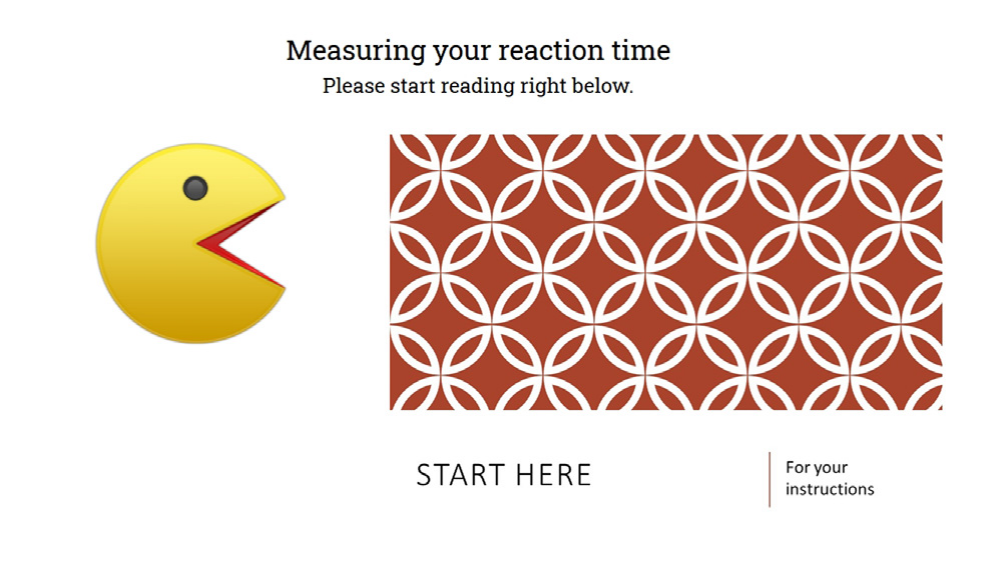
Webpage for the pre-conditions.

The pre-conditions were displayed on separate WordPress webpages (version 4.9.7) containing information on playing a Pac-Man game, see Figure 2 below. The no stress webpage had a hyperlink to a regularly functioning Pac-Man version that had been uploaded to a GitHub site. The stress webpage contained a hyperlink to a second, invalidated Pac-Man version on GitHub. The invalidated Pac-Man version did not properly respond to the user's arrow key strokes in 30% of the occasions. Instead it went into another randomly chosen direction. This type of invalidation for the purpose of generating participant stress was inspired by the Affective Pac-Man solution from the study of Reuderink et al. (2009).

After the pre-conditional phase -that took on average 4 min-was rounded off, the participants were redirected to the main experiment.

### 2.4 Main experiment

This main experiment was run on a WordPress website (version 4.9.7) that contained the eHealth intervention on the left side of the webpage. The eHealth intervention was a PowerPoint® presentation with psycho-education material on positive psychology. The goal of the eHealth psycho-education intervention was to make users knowledgeable about positive psychology. Positive psychology focuses on the abilities of people and their potential to flourish. Several treatments against depression are based on positive psychology principles (Hayes et al., 1999). In addition, positive psychology and happiness are subjects that are of general human interest. As we reasoned, this topic would contribute to engage participants for our experiment. The self-guided eHealth intervention contained a combination of theory and exercises, including the remunerated “three good things exercise” and “best possible self-exercise” (Renner et al., 2014).

#### 2.4.1 User support

**Figure 3:**
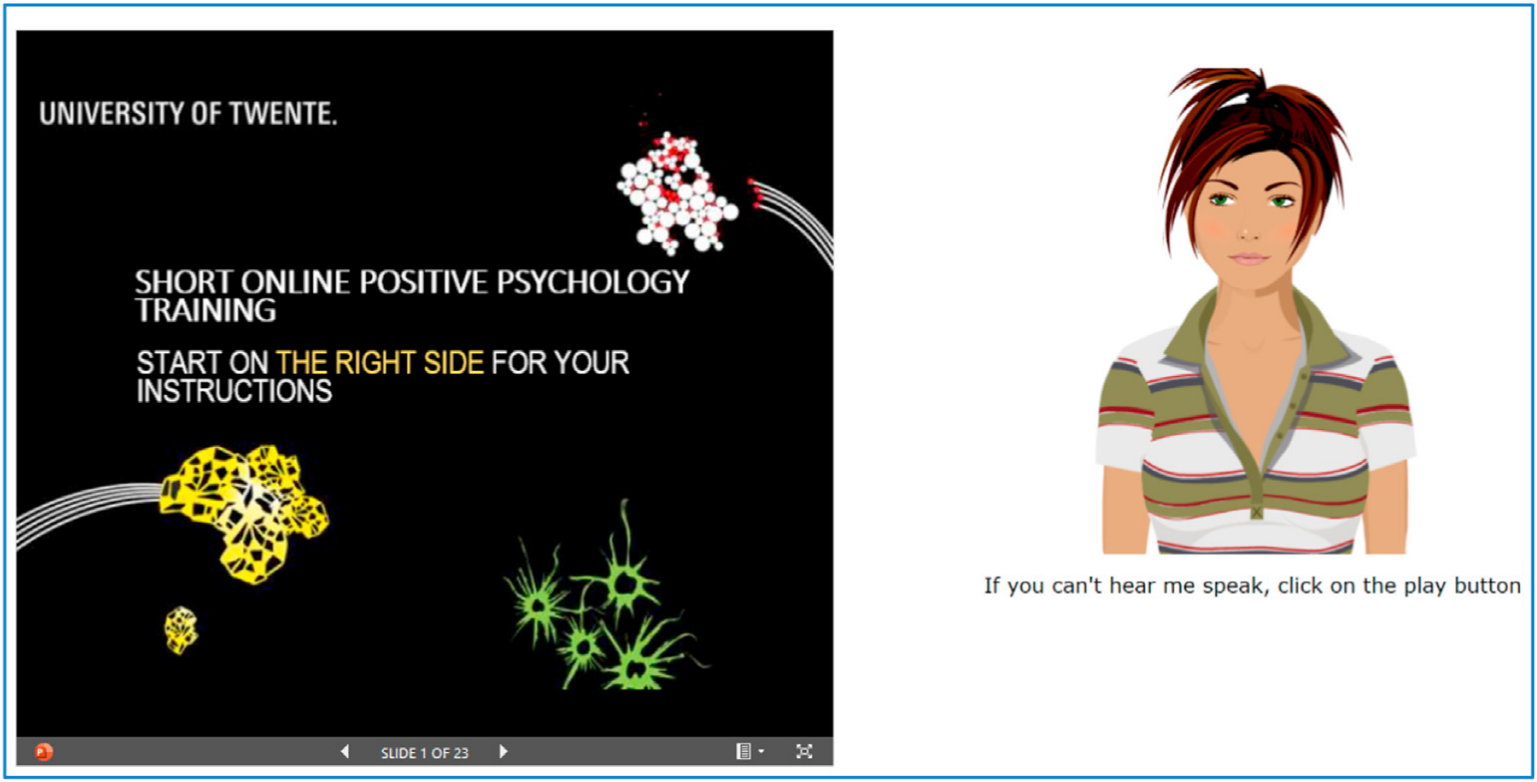
The eHealth psycho-education intervention. On the left side of the webpage the psycho-educational content is displayed, on the right side the support condition with guidance and directions (task-related support) and encouragement (motivational and emotional support) is presented. The example support condition shown is the female ECA, Eva.

As Figure 3 displays, user guidance and motivation were provided on the right side of the webpage by either a female ECA, male ECA or text in PowerPoint®. The female and male ECA conditions were created through the Voki application. The monologue-style ECA represented a virtual person in between 20 and 30 years of age, with Caucasian looks, acting as an informal (i.e. not medical) support provider. The user was asked to click on the ECA for the next voice segment to be spoken. The female and male voices were provided by two Dutch speakers. The ECA's showed lip synchronization and animation properties such as eyes blinking and chest breathing. Furthermore, the ECA's line of sight followed the cursor movements of the user. The textual guidance condition was created using Microsoft PowerPoint®. All support conditions expressed the same guidance conveyed in English. At the beginning, the ECA's uttered one additional phrase: “I am Eva/Brian, your virtual coach.” After each voice segment, the ECA's asked the user “Please click on the button to proceed.” And The guidance was a combination of task-related support (e.g., “within this experiment you will read about positive psychology and you will do some exercises”) and motivational support (e.g., “So, let's practice!”). In addition, the user was stimulated to take advantage of the exercises in daily life. The experiment took approximately 18 min for the participants to accomplish.

An explicit separation was created between the instructional phase during which the ECA (or text) provided instructions and the learning phase, following those instructions. This was done to control for the split-attention effect (Louwerse et al., 2005). The effect contends that an ECA that is starting up conversations will distract the student when he is processing the e-learning material. Therefore, during the leaning phase, the ECA was silent and placed in a different pane.

#### 2.4.2 Outcome measures

**Figure 4:**
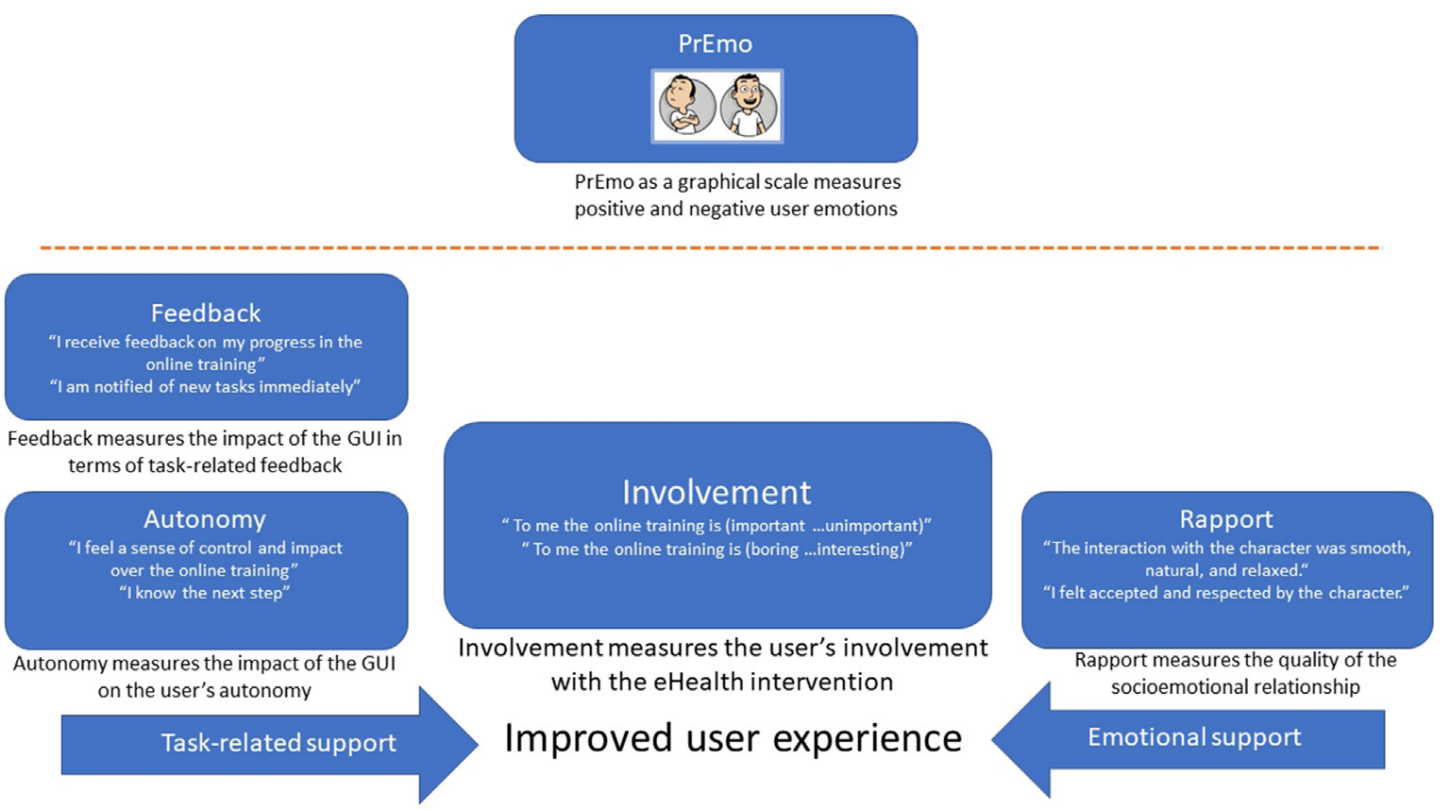
The questionnaires used. The ECA's task-related support (left side) and emotional support (right side) are hypothesized to have an impact on the user's involvement (middle) with the e-health intervention and on the positive and negative emotions (PrEmo) of the participant.

We selected a variety of outcome measures, to measure both the practical benefit of support (as presented on the left side of Figure 4) and the socio-emotional benefit (as presented on the right side of Figure 4). Furthermore, we expected both the practical and socio-emotional aspects to impact the key outcome variable (as presented in the middle); the user's involvement with the eHealth intervention. We also decided to explore the application of an innovative graphical outcome measure, PrEmo. All outcome variables are discussed in further detail below, going from left to right in Figure 4.

First, the autonomy and feedback dimensions of the larger EGameFlow scale (Fu et al., 2009) were selected as validated subscales that measure learners' enjoyment of e-learning games. Autonomy and feedback both represent the effects of task-related support. From both these scales three out of six items were chosen on the basis of validation and relevance to the experiment. Both scales use a seven-point Likert scale ranging from “strongly disagree” to “strongly agree.” Within the wording of the subscales the word “game” was replaced by “online training.”

Furthermore, as outcome variable involvement was selected. The Personal Involvement Inventory (Zaichkowsky, 1994) is a context free measure applicable to involvement with products, with advertisements, and with purchase processes. It has proven to be a useful outcome variable to evaluate environments with ECA's (Lo and Cheng, 2010; Scholten et al., 2019). In our case it assesses user motivation for the eHealth intervention. The scale consists of 10 items and uses a seven-point Likert scale with varying category names such as “important” vs. “not important” and “boring” vs. “interesting”. As indicated by the left and right arrows pointing to the middle, we expected that the involvement -as our ultimate outcome-with the eHealth intervention would be positively impacted by both the practical and motivational benefits of the ECA's support.

**Figure 5:**
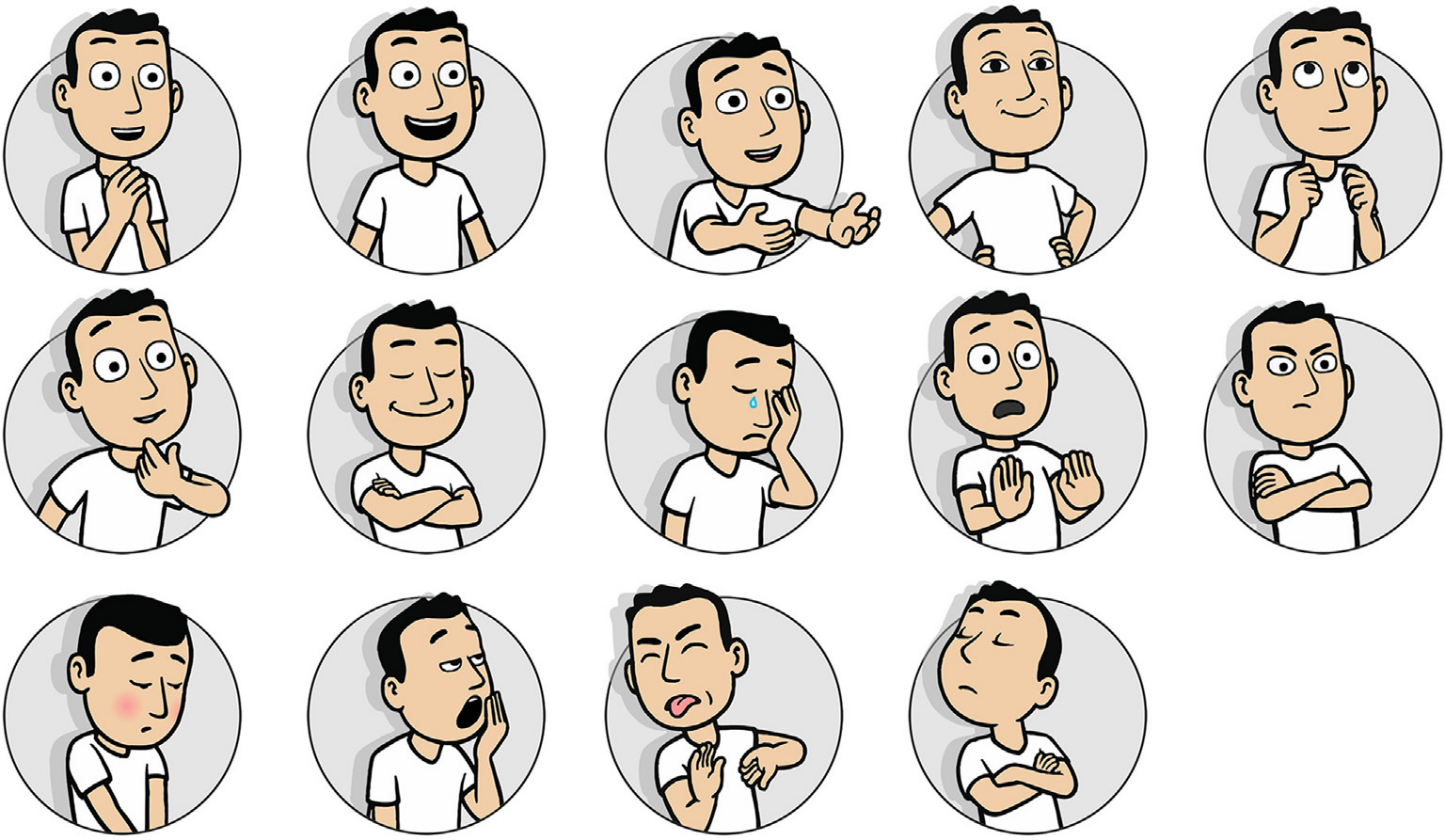
PrEmo visual outcome measure.

In addition, as outcome measure, PrEmo (Desmet et al., 2007) was chosen. PrEmo (see Figure 5 below) is a non-verbal self-report instrument that measures seven positive (further referred to as ‘PrEmoPos’) and seven negative emotions (further referred to as ‘PrEmoNeg’). It measures distinct emotions in a direct manner as it does not require the respondents to verbalize them. According to the developers (Desmet et al., 2007) and confirmed by Norman (2003) PrEmo offers important practical advantages when working with non-English speaking participants or other groups of people who might have difficulty verbalizing their feelings.

Last, the Rapport scale was selected. Rapport is an umbrella term for generic positive interactions between human counterparts, which as a term is also associated to terms as synchrony and flow. It has been used for ECA evaluations before (Brixey and Novick, 2019; Gratch et al., 2013). The Rapport scale (Cerekovic et al., 2014) consists of fifteen items of which we used fourteen. A question containing the term “Self-conscious” was left out for being too ambiguous. We rephrased the term “interaction” in “contact” and we used a seven-point Likert scale ranging from [(1) – Disagree strongly to (7) – Agree strongly].

#### 2.4.3 Procedure

The webpages were put online, and the study was run without human supervision to simulate self-guidance. Users were recruited from a pool of students of the university of Twente using the SONA system. Within the SONA systems, users could choose from an extensive list of experiments of which a portion was carried out online. Users had done similar experimental tasks before and we expected no differences amongst the users resulting from the number of SONA experiments they had previously participated in. Users were provided with an URL that led to the Qualtrics system. Within in the Qualtrics system they provided consent. Subsequently, they were directed to one of two pre-condition Wordpress webpages representing the stressful and no stress pre-conditions. After carrying out the pre-condition, users were led to a Qualtrics environment where users were asked to fill in their Pac-Man high scores. In addition, they were asked to fill in a short version of the PrEmo questionnaire as a check on the effectiveness of the pre-conditions (manipulation check). After that, users were re-directed to the e-psycho-education environment in Wordpress. During instruction on the right side of the webpage, the user was told what learning module would come next. Then the user was asked to click on the left side of the webpage and follow up on the psycho-education tasks. When the psycho-education module had come to an end, the user was asked to go to the right side of the webpage for new instructions. When three psycho-education modules were done, the user was re-directed to the Qualtrics environment where the final questionnaires were presented. After providing their answers, they and were rewarded with a course credit in the SONA system.

### 2.5 Data analysis

#### 2.5.1 Data distribution check

Before starting with the core statistical analysis, we first performed a check on the normality of the distributions of the outcome data. It appeared that the PremoNeg outcome variable was strongly left skewed (skewness = 2.3, kurtosis = 4.7). We tried to resolve this through re-normalization and by deleting outliers. However, both methods did not resolve the issue in a satisfactory way, so we decided not to involve PremoNeg in our main statistical analyses. Instead, we decided to run a seperate and specific non-parametric analysis on PremoNeg. In addition, the outcome variable Involvement showed some right skewness, (skewness = -1.2, kurtosis = 1.8), which we resolved (skewness = -0.7, kurtosis = 0.4) by deleting 3 outliers. As a result, the number of participants decreased from 106 to 103.

#### 2.5.2 Stress pre-conditions and guidance conditions

As a first step, we did a manipulation check on the effect of the stress and no stress pre-conditions as measured after the start and after the end of the experiment by means of the outcome variable PrEmo. For the PrEmoNeg outcome variable we applied a non-parametric test that suited the non-gaussian distribution of the data. For the normally distributed PrEmoPos outcome variable we utilized the t-test. Secondly, we grouped all the cases and divided them according to the following three factors:

1) ECA with a gender that matches the gender of the participants
2) ECA with a gender that does not match with the gender of the participants
3) Textual guidance and support (control variable)

and analyzed the differential effects on our outcome variables using ANOVA.

Thirdly, we analyzed the three guidance factors while taking stress into account using a two-way ANOVA. Furthermore, we performed a multiple regression test on the outcome variable involvement in order to find out to what extent external user support has an impact on user involvement.

## 3 Results

### 3.1 Manipulation check: stress effects at start and end of the experiment

To check whether we succeeded in inducing an efficient dose of stress amongst participants, we analyzed the PrEmo questionnaire as applied immediately after the pre-conditions. This questionnaire contained three items out of the six emotions for both PremoPos and PremoNeg. Furthermore, we analyzed the full PrEmoPos and PrEmoNeg questionnaire as applied after the experiment. The means, 95% Confidence Interval and SD values of the outcome variables are shown in Table 1.

**Table 1:** Mean scores and standard deviation of the effect of the pre-condition on the PrEmoNeg and PrEmoPos outcome variables, measured after the pre-conditions and after the experiment.

	Stress pre-condition (n = 53)	No stress pre-condition (n = 50)
PrEmoNegStart (1–5)∗	2.2 (1.9–2.5; 0.2)	1.9 (1.6–2.2; 0.2)
PrEmoNegEnd (1–5)∗	1.6 (1.3–1.9; 0.1)	1.8 (1.5–2.1; 0.2)
PrEmoPosStart (1–5)∗∗	2.7 (2.4–3.0; 0.1)	2.9 (2.6–3.2; 0.1)
PrEmoPosEnd (1–5)∗∗	3.0 (2.8–3.3; 0.1)	3.0 (2.7–3.3; 0.1)

∗ significant evolvement effect of p = 0.00.

∗∗ significant evolvement effect of p = 0.05.

The independent samples median test showed no significant differences between the stress and no stress pre-conditions PrEmoNegEnd (F = 1.22; p = 0.27); PrEmoPosStart (F = 1.01; p = 0.32); PrEmoPosEnd (F = 0.02; p = 0.88) although PrEmoNegStart showed some tendency (F = 3.10; p = 0.08).

As a follow-up analysis, we investigated how the PrEmo measurements had evolved from start to end during the experiment. For the no-stress pre-conditions we didn't find significant effects PrEmoPos (F = 0.39; p = 0.53); PrEmoNeg (F = 0.02; p = 0.89). However, for the stress pre-condition we found significant evolvement effects, strongest for PrEmoNeg (F = 10.50; p = 0.00), and lighter for PrEmoPos (F = 3.80; p = 0.05). Altogether, the evolvement results indicated that our initial stress manipulation had been effective and that stress had vanished at the end of the experiment.

#### 3.2 Stress vs. No stress

As a next step, we ran a one-way ANOVA to analyze the differences between the stress and no stress conditions. The means, 95% Confidence Interval and SD values of the outcome variables are shown in Table 2.

**Table 2:** Mean scores and standard deviation on the stress-no stress distinction.

	Stress pre-condition (n = 53)	No stress pre-condition (n = 50)
Feedback (1–7)	4.5 (4.2–4.8; 0.2)	4.7 (4.3–5.0; 0.2)
Autonomy (1–7)	5.2 (4.9–5.5; 0.2)	5.3 (5.0–5.7; 0.2)
Involvement (1–7)	5.2 (4.9–5.5; 0.2)	5.2 (4.9–5.5; 0.2)
PrEmoPos (1–5)	3.0 (2.7–3.3; 0.1)	3.0 (2.7–3.3; 0.1)
Rapport (1–7)	4.7 (4.4–5.1; 0.2) n = 35	4.6 (4.3–5.0; 0.2) n = 31

The one-way ANOVA revealed no significant differences on any of the outcome variables feedback (F=0.56; p=0.46), autonomy (F=0.36; p=0.55), involvement (F=0.00; p=0.99), PrEmoPos (F=0.02; p=0.88), and rapport (F=0.16; p=0.69).

#### 3.3 Stress and guidance

We sub-divided the stress and no stress pre-conditions into ECA guidance with a matching gender, ECA guidance with a not matching gender and finally textually guided section, in order to analyze potential differential effects between guidance/support conditions. The means, 95% Confidence Interval and SD values of the outcome variables are shown in Table 3.

**Table 3:** Mean scores and standard deviation on the stress/not-stress pre-condition distinction, subdivided into the three guidance and support conditions.

	Stress pre-condition (n = 53)			No stress pre-condition (n = 50)		
	A ECA matching gender (n = 12)	B ECA not matching gender (n = 23)	C Text (n = 18)	D ECA matching gender (n = 15)	E ECA not matching gender (n = 16)	F Text (n = 19)
Feedback (1–7)	5.0 (4.3–5.7; 0.4)	4.3 (3.8–4.8; 0.3)	4.5 (3.9–5.0; 0.3)	4.5 (3.9–5.1; 0.3)	5.3 (4.7–5.9; 0.3)∗	4.3 (3.8–4.9; 0.3)∗
Autonomy (1–7)	5.4 (4.7–6.0; 0.3)	5.2 (4.7–5.6; 0.2)∗∗	5.2 (4.7–6.0; 0.3)	5.5 (4.9–6.1; 0.3)	5.7 (5.2–6.3; 0.3)∗,∗∗	4.9 (4.4–5.4; 0.3)∗
Involvement (1–7)	5.0 (4.4–5.6; 0.3)	5.1 (4.7–5.5; 0.2)	5.4 (4.9–5.9; 0.3)	5.6 (5.0–6.2; 0.3)	4.7 (4.1–5.2; 0.3)	5.3 (4.8–5.7; 0.2)
PrEmoPos (1–5)	3.1 (2.5–3.7; 0.3)	3.1 (2.6–3.5; 0.2)	3.0 (2.5–3.5; 0.2)	2.9 (2.4–3.5; 0.3)	3.0 (2.5–3.5; 0.3)	3.1 (2.6–3.6; 0.2)
Rapport (1–7)	4.5 (4.0–5.1; 0.3)	4.8 (4.4–5.2 0.2)	n.a.	4.9 (4.4–5.4; 0.3)	4.4 (3.9–4.9; 0.2)	n.a.

∗ significant effect of p = 0.02.

∗∗ significant effect of p = 0.01.

Overall the two-way ANOVA showed no significant effects; autonomy (F = 1.30, p = 0.28); involvement (F = 1.42, p = 0.22); PrEmoPos (F = 0.06, p = 0.99); rapport (F = 0.99, p = 0.40), although feedback (F = 1.92, p = 0.10) showed some tendency. However, pairwise comparisons on individual conditions showed significant effects; for both feedback and autonomy the difference between E (no stress ECA not matching gender) and F (no stress text) was significant (in both cases p = 0.02) in the direction of the ECA. Furthermore, for feedback the difference between B (stress ECA not matching gender) and E (no stress ECA not matching gender) was significant (p = 0.01) in the direction of the no stress pre-condition.

#### 3.4 Effect of a gender match between ECA and participant

Next, we grouped the stress and no stress cases together and analyzed the effect of a match of gender between the participant and the ECA, using text as a control variable. The means, 95% Confidence Interval and SD values of the outcome variables are shown in Table 4.

**Table 4:** Mean scores and standard deviation on the gender match/mismatch distinction.

	Matching gender (n = 27)	Not matching gender (n = 39)	Text (n = 37)
Feedback (1–7)	4.7; 4.3–5.2; 0.2	4.7; 4.3–5.1; 0.2	4.4 (4.0–4.8; 0.2)
Autonomy (1–7)	5.0; 4.7–5.4; 0.2	5.4; 5.0–5.8; 0.2	5.0 (4.7–5.4; 0.2)
Involvement (1–7)	5.3; 4.9–5.7; 0.2	4.9; 4.6–5.3; 0.2	5.3 (5.0–5.7; 0.2)
PrEmoPos (1–5)	3.0; 2.6–3.4; 0.2	3.0; 2.7–3.4; 0.2	3.0 (2.7–3.4; 0.2)
Rapport (1–7)	4.7; 4.3–5.1; 0.2	4.6; 4.3–4.9; 0.2	n.a.

The one-way ANOVA revealed no significant differences on any of the outcome variables feedback (F = 0.70; p = 0.50), autonomy (F = 1.48; p = 0.23), involvement (F = 1.61; p = 0.21), PrEmoPos (F = 0.00; p = 0.99) and rapport (F = 0.13; p = 0.72). As a next step, we left out the textual condition and sub-divided the matching gender conditions in a female and male participant section, in order to analyze potential differential matching gender effects between ECA and user. We ran a two-way ANOVA. the means, 95% Confidence Interval and SD values of the outcome variables are shown in Table 5.

**Table 5:** Mean scores and standard deviation on the gender match/mismatch distinction subdivided to the participant's gender.

	Matching gender (n = 27)		Not matching gender (n = 39)	
	A Female participant (n = 22)	B Male participant (n = 5)	C Female participant (n = 27)	D Male participant (n = 12)
Feedback (1–7)	4.6; 4.1–5.2; 0.3	5.2; 4.0–6.4; 0.6	4.7; 4.2–5.2; 0.3	4.7; 3.9–5.5; 0.4
Autonomy (1–7)	5.3; 4.8–5.7; 0.2	6.2; 5.3–7.1; 0.5	5.4; 5.0–5.8; 0.2	5.4; 4.8–6.0; 0.3
Involvement (1–7)	5.5; 5.0–6.0; 0.2∗	4.6; 3.0–5.6; 0.5	5.1; 4.7–5.6; 0.2	4.4; 3.8–5.1; 0.3∗
PrEmoPos (1–5)	3.1; 2.6–3.5; 0.2	2.9; 1.9–3.9; 0.5	3.0; 2.5–3.4; 0.2	3.2; 2.6–3.9; 0.3
Rapport (1–7)∗∗∗	4.9; 4.5–5.3; 0.2∗∗	4.2; 3.3–5.0; 0.4	4.9; 4.5–5.3; 0.2∗	4.1; 3.5–4.6; 0.3∗,∗∗

∗ significant pairwise effect of p = 0.01.

∗∗ significant pairwise effect of p = 0.02.

∗∗∗ significant ANOVA effect of p = 0.04.

We found an effect from the ANOVA for rapport (F = 2.98; p = 0.04) indicating that the four conditions differed significantly from each other. For the other outcome variables we did not find a significant effect; feedback (F = 0.95; p = 0.42), autonomy (F = 1.01; p = 0.39), PrEmoPos (F = 0.16; p = 0.92) although involvement showed a strong tendency (F = 2.60; p = 0.06).

Pairwise comparisons for involvement showed significant differences between female participants with a matching gender (A) and male participants with a non-matching gender (D) (p = 0.01) in the direction of A. Pairwise comparisons for rapport demonstrated two effects; first a significant difference between female participants with a matching gender (A) and male participants with a non-matching gender (D) (p = 0.02) in the direction of A. Second, a significant difference between female participants with a non-matching gender (C) and male participants with a non-matching gender (D) (p = 0.01) in the direction of C. So, male participants of a non-matching gender (D) showed significantly lower rapport scores than female participants (A and C), irrespective of their gender match.

#### 3.5 The potential role of the participant's gender

As a next step, we analyzed the effect of the participant's gender (female versus male) as a stand-alone factor by a one-way ANOVA. Our objective was to find out whether ECA guidance had a differential effect on female versus male participants as the previous results had suggested. The means, 95% Confidence Interval and SD values of the outcome variables are shown in Table 6.

**Table 6:** Mean scores and standard deviation on the participant's gender distinction.

	Female participant (n = 49)	Male participant (n = 17)
Feedback (1–7)	4.7; 4.3–5.0; 0.2	4.8; 4.2–5.5; 0.3
Autonomy (1–7)	5.3; 5.0–5.6; 0.1	5.6; 5.1–6.1; 0.3
Involvement (1–7)∗	5.3; 5.0–5.6; 0.2	4.5; 3.9–5.0; 0.3
PrEmoPos (1–5)	3.0; 2.7–3.3; 0.2	3.1; 2.6–3.7; 0.3
Rapport (1–7)∗∗	4.9; 4.6–5.1; 0.1	4.1; 3.6–4.5; 0.2

∗ significant effect of p = 0.01.

∗∗ significant effect of p = 0.00.

We found a significant effect for involvement (F = 6.66; p = 0.01), and for rapport (F = 9.14; p = 0.00) all in the direction of female participants. On the other variables no effects were found: feedback (F = 0.25; p = 0.62), autonomy (F = 1.10; p = 0.30), and PrEmoPos (F = 0.13; p = 0.72).

#### 3.6 The potential role of the ECA's gender

As a next step, we analyzed the effect of the ECA's gender (female versus male) as a stand-alone factor by a one-way ANOVA. Our objective was to find out whether guidance by either a female ECA or male ECA had a differential effect on participants. The means, 95% Confidence Interval and SD values of the outcome variables are shown in Table 7.

**Table 7:** Mean scores and standard deviation on the participant's gender distinction.

	Female ECA (n = 34)	Male ECA (n = 32)
Feedback (1–7)	4.7; 4.2–5.1; 0.2	4.8; 4.3–5.2; 0.2
Autonomy (1–7)	5.3; 5.0–5.7; 0.2	5.5; 5.2–5.9; 0.2
Involvement (1–7)	5.1; 4.7–5.5; 0.2	5.1; 4.6–5.5; 0.2
PrEmoPos (1–5)	3.1; 2.7–3.5; 0.2	3.0; 2.6–3.3; 0.2
Rapport (1–7)	4.6; 4.2–4.9; 0.2	4.8; 4.4–5.1; 0.2

With respect to the gender of the ECA, no significant effects were found; feedback (F = 0.11; p = 0.75), autonomy (F = 0.60; p = 0.44), involvement (F = 0.05; p = 0.82), PrEmoPos (F = 0.32; p = 0.58), rapport (F = 0.76; p = 0.39).

#### 3.7 User involvement and external support

Finally, we conducted a multiple regression test in order to check the extent to which user involvement was impacted by task-related support and emotion-related support. We took the outcome variable involvement as the dependent variable and feedback, autonomy and rapport as independent variables. The resulting regression was significant and explained 32% of variance (R-squared 0.32; F = 29.54; p = 0.00). Significant predictor in the model was rapport (t = 5.4; p = 0.00) but not feedback (t = 0.40; p = 0.71) and autonomy (t = 0.00; p = 0.95). Our expectation that both types of support would contribute to user involvement was therefore not confirmed.

## 4 Discussion

### 4.1 Principal results

We found one out of our hypothesized effects: the guidance of the monologue-style ECA yielded higher scores than textual guidance, but only in the no stress conditions. Such was the case for the feedback and autonomy outcome variables and this was a replication of the positive ‘ECA guidance compared to text effect’ of the Scholten et al. (2019) study. Contrary to our hypothesis, our way of inducing stress did not positively impact the user's evaluation of the ECA. Instead, it neutralized the positive ECA effect. Thirdly, a main effect of a matching gender between participant and ECA was not found. Relevant interaction effects were found, though. For involvement and rapport effects were found of female participants with a matching gender that scored significantly higher than their male counterparts with a not matching gender. Following from the previous outcomes, our fourth hypothesis of higher involvement with the eHealth intervention resulting from the deployment of the ECA under stressful circumstances, was not confirmed. Not hypothesized, we found a prominent effect of the gender of the participant interacting with the ECA; female participants scored significantly higher than their male counterparts on both involvement and rapport.

### 4.2 Interpretation of our effects

*No ECA* support *effect compared to text as the control condition* Regarding guidance, we found that there was no preference for the ECA compared to text within the stressful conditions. It is known from the social psychology literature that the attention of others fosters mastery of simple tasks but impairs mastery of complex and stressful tasks. This is also known as the theory of social facilitation and inhibition (Steinmetz and Pfattheicher, 2017; Zajonc and Sales, 1966). With the ECA study field, these impairment effects have been previously reported on; female participants were hindered by the presence of the ECA when they performed a task that was stressful due to its novelty (Zanbaka et al., 2004). Furthermore, Rickenberg and Reeves (2000) found that users felt more anxious when an ECA monitored their website work which led to a decrease in the user's task performance. Note however, that within our experiment we deployed the ECA with the explicit goal of facilitating users. The ECA introduced itself as a support provider and as such we expected user facilitation instead of hindering. Furthermore, within our ECA design, we separated the main eHealth intervention from the guidance part as to control for the distraction effect. However, these precautions appeared not so preserve the ECA's facilitating effect under stressful conditions. A potential explanation is the continuous visibility of the ECA, including the phase that the participants carried out their tasks. This leads to the follow-up question what kind of ECA set-up could have been more appropriate. We think of additional support more explicitly pointing to the user experience of stress, potentially stated prior to the stressful event; e.g. “During the coming event you will potentially experience some stress, that is not uncommon. Note that I will be here after this event to guide you through the course.” Secondly, stressful users potentially differ in their preferences. Users experiencing stress could be given the option to be either guided by textual information or an ECA. In other words, support could be personalized. A third option to consider, is that textual guidance may be an equal or even the more suitable solution in stressful contexts. This option is in accordance with the results Ranjbartabar and Richards (2018) who showed that rapport with the textual control condition was higher than that of their two ECA groups, although the effect did not reach significance. In short, the strength of ECA's is that they are more human-like, but this can potentially turn out to be a weakness in situations in which users wish to act on an autonomous basis.

#### 4.2.1 Stress did not induce higher ECA evaluations

In our experiment, our implementation of stress did not initiate the hypothesized causal chain of a higher user need for support and therefore more elevated levels of appreciation for the ECA. In order to understand the absence of this effect, we first checked whether we had been successful in inducing stress on participants in the first place. The analysis on the evolvement of positive and negative emotions both showed a significant improvement from start to end of the experiment in case of the stressful pre-condition. These results suggested that the stress manipulation had been successful.

Moreover, for all pre-conditions, the evolvement of all the PrEmo variables showed that users had lost their stress at the end of the experiment. This sketches an image of participants who are nervous at start of the experiment, just after they have played the pre-conditional Pac-Man game. During the experiment the participants recovered from the stress, possibly with some help of the Positive Psychology intervention. From other studies, we know that the duration of emotions including stress are highly variable, with emotions lasting anywhere from a few of seconds up to several hours, or even days (Verduyn et al., 2009). We reckon that either a stronger single dose of stress or multiple doses of stress during the experiment could have kept the participants in a more prolonged stressed state. Furthermore, inducing stress is not very common within eHealth experiments and is of course bounded by ethical guidelines. Moreover, Sponselee et al. (2004) mention that artificial stressful tasks are very difficult to set up for experimentation purposes. She therefore recommends the application of natural stressors (e.g. selecting students directly after their exams). Altogether, we recommend further research with regards to the induction of stress, measuring stress levels and the potential relief through computerized support.

#### 4.2.2 No higher ECA evaluations when the ECA's gender matches with the participant

With regards to a gender match between ECA and participant we did not find a main effect. As an explanation for the absence of a main matching gender effect, the literature (e.g. ter Stal et al., 2019) mentions that although (gender) resemblance between participant and user is an important factor, multiple factors should be taken into consideration. This includes the ECA's age, voice and its role. Within our set-up there was no difference between the female ECA and male ECA with regards to their ages and roles. In both cases we applied an ECA in its twenties, taking the role of a support provider. So this will not provide an explanation. As a result, we can only think of a very complex explanation such as the voice quality of the male ECA that outperformed the voice quality of the female ECA, but only in the eyes of female participants. Note also that the absence of a gender match effect is not unprecedented. Other studies (e.g., Richards et al., 2020) have reported on a matching gender that appeared not to have an effect.

#### 4.2.3 Effect of the participants' gender

Our study demonstrated higher involvement and rapport among female participants than among male participants. This is not unprecedented. Foster (2007) has reported on female users who responded more positively to expressive embodied agents than male users. However, we find it hard to generalize the finding of the Foster's study and ours; more research on this subject is needed.

#### 4.2.4 User involvement can partially be ascribed to user support

Our regression analysis showed that a portion of the user involvement could be ascribed to emotion-related support, i.e. rapport. The regression analysis did not show a positive effect of feedback, neither of autonomy on the user's involvement with the eHealth intervention. This finding suggests that although feedback and autonomy can be helpful for users (e.g. know how to navigate smoothly) this doesn't translate into enlarged user involvement.

## 5 Conclusion

Our main ambition for studying the effectiveness of a monologue-style ECA acting as an adjunct to a self-guided eHealth context was its potential to deliver higher evaluated user guidance and support than plain text. However, our experimental results demonstrated that our ECA only succeeded in outperforming text under not stressful circumstances, in line with the results of our earlier study. This lack of evidence is not unprecedented in the ECA study field. As has been put forward within several ECA review studies; ECA research is multi-faceted and experimental studies regularly provide mixed and inconclusive results. We consider the results of our study as an affirmation of this phenomenon. Moreover, we realize that ECA research is challenging. The implementation of the ECA has to be spot-on for the participant to accept and prefer the ECA over textual guidance. If it is not implemented precisely right, the ECA will not yield preferential effects. In our study involving stress, the visibility of the ECA during task completion -despite its silent state-could have contributed to the absence of preferential effects for the ECA. This means that an alternative set-up for the delivery of support has to be considered. We think that the option of textual guidance and support should be re-evaluated. Text may sometimes do the job as well as a monologue-style ECA. With regards to this study's purpose, once more we emphasize the widely prevalence of patient stress and the potential from the eHealth technology itself to offer relief and support. Investigating the effectiveness of stress-relief by persuasive technology is relevant and in our eyes merits future research. However, as the literature describes, research on stress-induction and computerized relief is probably as complex as the ECA study field, so definitely more research is needed here. However, we have an important objective. The moment that we have succeeded in deploying effective stress-reducing technology, many eHealth users around the world will benefit and lead better lives.

## 6 Limitations

Conclusions on ECA research are specific to their task and context. Concerning the task and context that were used in our experimental set-up and that could have influenced our results; we separated learning content (left part of the screen) from supportive content (right part of the screen). In addition, as learning content we used a positive psychology intervention.

As supportive content we provided directions and gave positive feedback after a learning task was finalized by the user. This way we avoided direct distraction from the ECA toward the user. However, the visual presence of the ECA during task completion was not controlled for.

The supportive content could be controlled by the user by using the forward and backward buttons. This provided user control but deviated from other ECA set-ups that use vocal user input. Our intervention was a short-term, one-off intervention. It is not known how this can be translated to life interventions that typically span a period of 6–10 weeks and are used on a more frequent basis.

The manner we induced stress, the invalidated PacMan solely resulted in partial effects and is of course just an experimental representation of what chronic patients experience using eHealth solutions.

## 7 Directions for future work

Future research can be carried out by creating set-ups that contain optional guidance by either text or an ECA that can be invoked by the user, at different moments in time during the experiment. After providing guidance and support, this facility will disappear again. By alternating stressful and not stressful tasks, it can be more deeply investigated which support and guidance option (if any) is preferred by the user in which situation. Furthermore, by the creation of an invocation mechanism with a short duration, the ECA's (or supportive text) are no longer visible during the episodes that the participants do their tasks and potential task inhibition can be avoided. Moreover, a personalized set-up can be realized this way. By deploying a modern version of the rapport questionnaire (see Ranjbartabar and Richards, 2018), the distinctive effects between textual guidance and ECA guidance on relationship building can be assessed. In addition, a naturally appearing stressful event could be involved in the experimental set-up, such as students who have just finalized their exams. Furthermore, measurement of stress can be done by deploying smart devices such as wristbands (see e.g. Sevil et al., 2017) in order to track the stress' temporal dynamics. Subsequently, these measurements can be cross-validated with questionnaires on stress experiences as presented at the end of the experiment. With regards to the ECA's credibility and effectiveness, the ECA can tell that there is a chance that there will be difficult episodes for the user, prior to the experiment. This can enlarge the credibility of the ECA, that can potentially be helpful at a later stage when the user truly experiences frustration and has te opportunity to ask for external support. Last, we know what users are confronted with during the stressful PacMan and neutral PacMan pre-conditions. Without the need of measuring the user's emotions, the ECA can provide a higher dose of empathy towards the users who have just experienced the malfunctioning PacMan. All these possible directions, should be implemented with precision. As the varying and often contradictory results within the ECA study field have demonstrated, the context matters a lot and so do the details of the design of the support.

## Declarations

### Author contribution statement

M. R. Scholten: Conceived and designed the experiments; Performed the experiments; Analyzed and interpreted the data; Contributed reagents, materials, analysis tools or data; Wrote the paper.

S. Kelders, J. E. W. C. Van Gemert-Pijnen: Conceived and designed the experiments; Analyzed and interpreted the data; Wrote the paper.

### Funding statement

This research did not receive any specific grant from funding agencies in the public, commercial, or not-for-profit sectors.

### Data availability statement

Data included in article/supplementary material/referenced in article.

### Declaration of interests statement

The authors declare no conflict of interest.

### Additional information

No additional information is available for this paper.

## Appendix A Supplementary data

Submitted Data Set Dec 9 2020 Submitted Data Set Dec 9 2020

Coaching Script Main Exp April 3 2019 Coaching Script Main Exp April 3 2019

Descriptive Statistics Experiment Descriptive Statistics Experiment
