# Supplementary material for: Can a monologue-style ECA more effectively motivate eHealth users in initial distress than textual guidance?
Source: Heliyon. 2021 Mar 21;7(3):e06509. doi: 10.1016/j.heliyon.2021.e06509 (PMC8020434; doi:10.1016/j.heliyon.2021.e06509)
Supplement: Coaching Script Main Exp April 3 2019 [file mmc2.docx]

**1.**I am **Eva / Brian** your Virtual Coach.

Welcome to the main experiment.
This experiment is about online training of positive psychology.
Please click on the spacebar to proceed.

**2.**On the left side, you find a short training on positive psychology.
You will read some information and do some exercises.
I am here to guide you through the training.
Please click on the spacebar to proceed.

**3.**This experiment will take you about 20 to 30 minutes, during which you will learn some interesting facts about positive psychology.
You will also do some exercises.
At the end we will ask you some questions about your experiences.
Please click on the spacebar to proceed.

**4.**Ok, let’s start.

Go to the PowerPoint on the left and read the theory on slides 1-13.
Then come back to me and click on the spacebar to proceed.

**5.**Ok, now you know that the PERMA model can contribute to your feelings of well-being and happiness. By applying the PERMA model knowledge in your daily life you can enlarge your feelings of well-being. So, let’s practice!

Go back to the PowerPoint on the left and do the three good things exercise.
Then come back to me and click on the spacebar to proceed.

**6.**Ok, what did you think about the three good things exercise?
It works best if you practice it every night for one or two weeks. And there are more valuable exercises such as the Best Possible Self exercise. Please go to the left to do it.
Then come back to me and click on the spacebar to proceed.

**7.**Did you like the Best Possible Self exercise?
If you want you can continue practicing with it at home.
In order to do se, reserve 10 minutes during the coming 7 days.

You can go beyond that, by creating another Best Possible Self.
Visualize yourself doing another challenging activity such as sports or playing a music instrument.
Please click on the spacebar to proceed.

**8.**Ok, let’s wrap up.
You have read information about positive psychology and you have done two exercises that can contribute to your feelings of well-being. If you feel they are valuable to you, you can continue with the exercises at home.

Don’t forget: practice makes perfect!
Please click on the spacebar to proceed.

**9.**Ok, it is time for you to let us know about your experiences.
You find a link below that leads to a few sets of questions.

Please click on the link below for a new window to open.
After answering the questions, you are done.
Thank you for your participation! Bye!
