# Supplementary material for: Can a monologue-style ECA more effectively motivate eHealth users in initial distress than textual guidance?
Source: Heliyon. 2021 Mar 21;7(3):e06509. doi: 10.1016/j.heliyon.2021.e06509 (PMC8020434; doi:10.1016/j.heliyon.2021.e06509)

DESCRIPTIVE STATISTICS EXPERIMENT

**Legend**
Number of participants (N)
Scale (Minimum up to Maximum)
Mean score (Mean)
Standard Deviation (Std. Deviation)

**Feedback and autonomy**


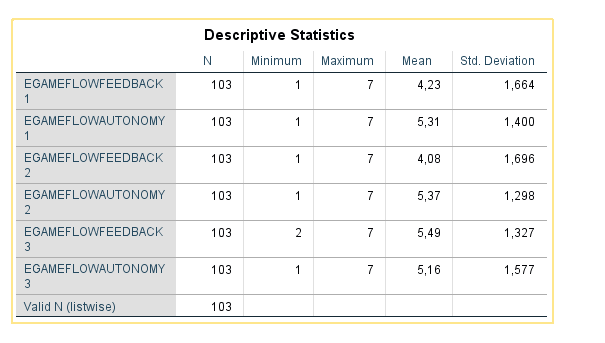


**Involvement**


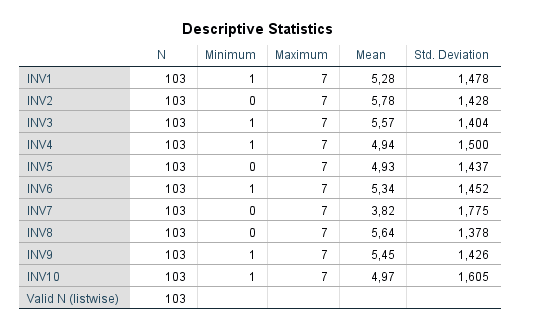


**Rapport**


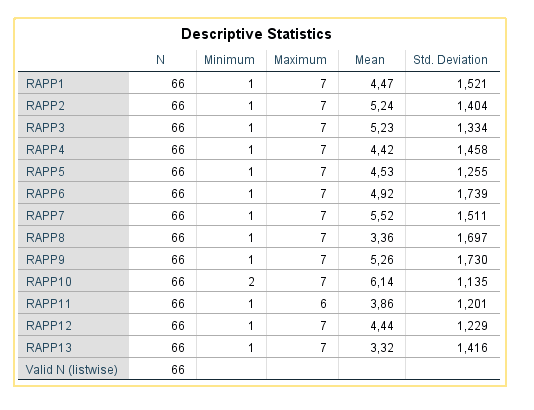


**PrEmo**


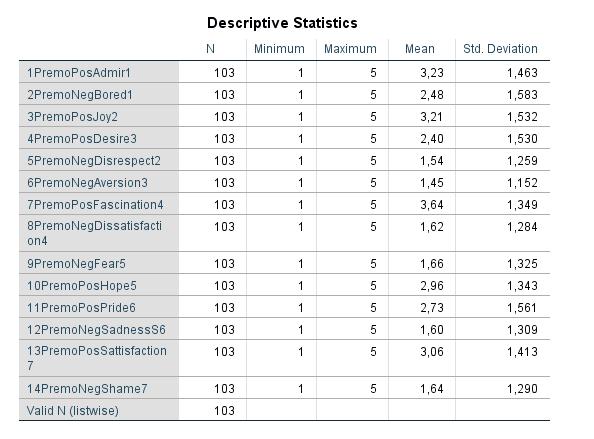

Supplement: Descriptive Statistics Experiment [file mmc3.docx]
